# Clinical impact of low-radiation computed tomography coronary angiography diagnosis for coronary artery stenosis: Study Protocol

**DOI:** 10.1097/MD.0000000000017474

**Published:** 2019-11-15

**Authors:** Jian-Jun Li, Ming Zeng

**Affiliations:** a Department of CT Diagnosis, Yan’an People's Hospital, Yan’an, China; b Department of Radiology, Yan’an Hospital of Traditional Chinese Medicine, Yan’an, China.

**Keywords:** coronary artery stenosis, low-radiation computed tomography coronary angiography, sensitivity, specificity

## Abstract

**Background:**

The objective of this study aims to assess the clinic impact of low-radiation computed tomography coronary angiography (LR-CTCA) diagnosis for coronary artery stenosis (CAS).

**Methods:**

This study will comprehensively search the following electronic databases from inception to the present: PUBMED, EMBASE, Cochrane Library, PsycINFO, Web of Science, Google, Allied and Complementary Medicine Database, Chinese Biomedical Literature Database, VIP database, WANGFANG, and China National Knowledge Infrastructure. All these electronic databases will be searched without language restrictions. All case-controlled studies on assessing the clinical impact of LR-CTCA diagnosis for patients with CAS will be included. Quality Assessment of Diagnostic Accuracy Studies tool will be utilized to evaluate the methodological quality for each qualified studies.

**Results:**

We will assess the clinic impact of LR-CTCA diagnosis for CAS by measuring sensitivity, specificity, positive likelihood ratio, negative likelihood ratio, and diagnostic odds ratio.

**Conclusion:**

The results of this study will summarize the latest evidence of LR-CTCA diagnosis for CAS.

**Systematic review registration:**

PROSPERO CRD42019139336.

## 1 Introduction

Coronary artery disease (CAD) is often associated with poor outcome results, and also a major cause of morbidity and mortality.^[1–4]^ It often manifests as chest pain, shortness of breath, and heart attack.^[5–7]^ Such disorder often results from blockage or stenosis of the coronary arteries that supply the blood to the heart.^[8–10]^ Of which, coronary artery stenosis (CAS) is the most common type, and it often occurs at the early stage of CAD.^[11–12]^ A variety of risk factors are relevant with this condition, such as age, sex, family history, smoking, high blood pressure, high blood cholesterol levels, diabetes, overweight or obesity, physical inactivity, high stress, and unhealthy diet.^[13–18]^ Thus, it is very important to prevent CAS with effective diagnosis tool at early stage. Low-radiation computed tomography coronary angiography (LR-CTCA) is reported to diagnose CAS at early stage accurately and effectively.^[19–35]^ However, its results are still inconsistent. Therefore, this study will firstly assess the accurate of LR-CTCA diagnosis for patients with CAS.

## 2 Methods

### 2.1 Study registration

The protocol of this study has been registered on PROSPERO (CRD42019139336). The study will be reported in accordance to the guideline of preferred reporting items for systematic reviews and meta-analysis (PRISMA) protocol statement.^[36]^

### 2.2 Ethics and dissemination

We will not analyze individual patient data, thus, no ethic approval is required. We will plan to publish results of this study via peer-reviewed journals or conference proceedings.

### 2.3 Eligibility criteria

#### 2.3.1 Types of studies

All case-controlled studies (CCSs) on assessing clinical value of LR-CTCA diagnosis for CAS will be considered for inclusion.

#### 2.3.2 Types of patients

We will include patients with invasive coronary angiography diagnosis of CAS in this study regardless the gender, age, and region.

#### 2.3.3 Type of index test

Index test: LR-CTCA diagnosis for CAS has been utilized in the intervention group.

Reference test: invasive coronary angiography diagnosis for CAS has been used in the control group.

#### 2.3.4 Types of outcome measurements

In this study, we will measure sensitivity, specificity, positive likelihood ratio, negative likelihood ratio, and diagnostic odds ratio.

### 2.4 Search methods for identification of studies

#### 2.4.1 Electronic searches

In this study, the databases of PUBMED, EMBASE, Cochrane Library, PsycINFO, Web of Science, Google, Allied and Complementary Medicine Database, Chinese Biomedical Literature Database, VIP database, WANGFANG, and China National Knowledge Infrastructure will be comprehensively searched from their inception to the present regardless language restrictions. We will consider all CCSs of LR-CTCA diagnosis for CAS. A detailed example of search strategy for PUBMED is presented in Table 1. We will adopt similar strategies to all other electronic databases.

**Table 1:**
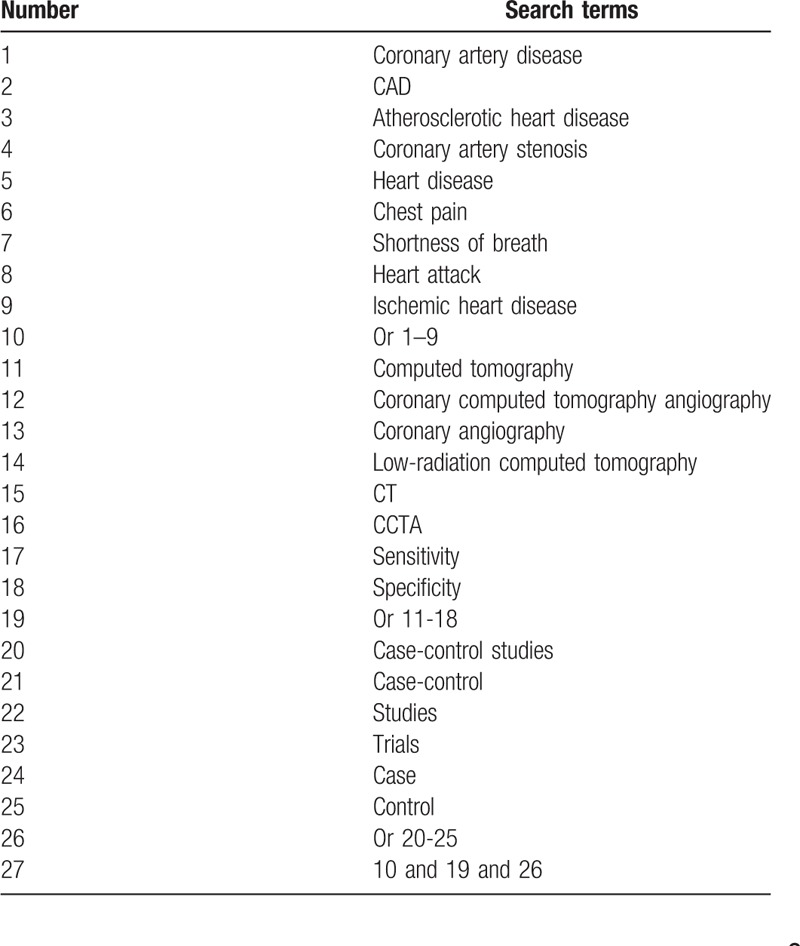
Search strategy for PUBMED database.

#### 2.4.2 Other resources

Other resources, such as dissertations, conference proceedings, and reference lists of relevant reviews will be included.

### 2.5 Data collection

#### 2.5.1 Selection of studies

Two independent authors will screen study eligibility according to the inclusion criteria. All divergences between 2 authors will be solved by consensus with a third author involved. The whole process consists of 2 stages. At first stage, all records will be scanned, and all duplicated and irrelevant studies will be excluded. At the second stage, full texts of the remaining studies will be read to further judge if they meet all eligibility criteria. All excluded studies will be recorded for specific reasons. The results of study selection will be shown in the PRISMA flow chart.

#### 2.5.2 Data collection and management

Two independent authors will extract data from all eligible studies based on the previously developed data extraction form. A third author will help to resolve all disagreements between 2 authors via discussion. The following information will be extracted:

(1) Study characteristics: first author, year of publication, region, design, study setting, sample size, and so on;
(2) Patient characteristics: patient demographics, inclusion and exclusion criteria, and so on;
(3) Interventional and reference tests: time, methods of samples, study period, and so on;
(4) Outcomes: true positives, false positives, and so on.

#### 2.5.3 Dealing with missing data

Whenever there is insufficient or missing data, we will directly contact primary authors of original studies to require those data. If we cannot get back those data, we will analyze the available data only.

### 2.6 Methodological quality assessment

Two independent authors will assess methodological quality for each qualified study using the Quality Assessment of Diagnostic Accuracy Studies tool.^[37]^ Any different opinions regarding the methodological quality assessment between 2 authors will be solved by a third experienced author through discussion.

### 2.7 Statistical analysis

RevMan V.5.3 and Stata 12.0 software will be used to carry out statistical analysis. We will calculate descriptive statistics and 95% confidence intervals. Additionally, we will also operate descriptive forest plot and a summary receiver operating characteristic plot.

#### 2.7.1 Assessment of heterogeneity

We will use *I*^2^ statistic to investigate heterogeneity among eligible studies. The value of *I*^2^ ≤ 50% means low heterogeneity. On the other hand, the value of *I*^2^ > 50% means significant heterogeneity.

#### 2.7.2 Data synthesis

If there is low heterogeneity (*I*^2^ ≤ 50%), we will pool the data and carry out meta-analysis. If there is significant heterogeneity (*I*^2^ > 50%), we will carry out subgroup analysis. We will pool the data and perform meta-analysis if there is low heterogeneity after subgroup analysis. Otherwise, we will not carry out meta-analysis if there is significant heterogeneity after subgroup analysis. In addition, we will carry out the bivariate random-effects regression to summarize the estimates of sensitivity and specificity.

### 2.8 Additional analysis

#### 2.8.1 Subgroup analysis

We will conduct subgroup analysis based on the different study characteristics, and patients.

#### 2.8.2 Sensitivity analysis

We will carry out sensitivity analysis to explore the stability and robustness of pooled outcome results by removing the low methodological quality studies.

#### 2.8.3 Reporting bias

We will perform funnel plots and relevant regression tests^[38]^ to check if there is reporting bias in this study.

## 3 Discussion

Although many studies utilized LR-CTCA diagnosis for CAS, there is no comprehensive systematic review comparing the diagnosis accurate of LR-CTCA with other diagnosis tools. We hope this study will provide the most current available evidence to present whether LR-CTCA diagnosis is more accurate than other diagnosis tools in the patients diagnosed as CAS. The results of this study will provide helpful evidence for the LR-CTCA diagnosis in patients with CAS. However, this study may still have several limitations. First, although this study tries to search literature records comprehensively, this study may still miss some potential studies. Second, different methodological qualities may cause high heterogeneity.

## Author contributions

**Conceptualization:** Jian-Jun Li, Ming Zeng.

**Data curation:** Jian-Jun Li, Ming Zeng.

**Formal analysis:** Jian-Jun Li, Ming Zeng.

**Investigation:** Ming Zeng.

**Methodology:** Jian-Jun Li.

**Project administration:** Ming Zeng.

**Resources:** Jian-Jun Li.

**Software:** Jian-Jun Li.

**Supervision:** Ming Zeng.

**Validation:** Jian-Jun Li, Ming Zeng.

**Visualization:** Jian-Jun Li, Ming Zeng.

**Writing – original draft:** Jian-Jun Li, Ming Zeng.

**Writing – review and editing:** Jian-Jun Li, Ming Zeng.
